# Identification of TMZ resistance‐associated histone post‐translational modifications in glioblastoma using multi‐omics data

**DOI:** 10.1111/cns.14649

**Published:** 2024-03-06

**Authors:** Liguo Ye, Lingui Gu, Yaning Wang, Hao Xing, Pengtao Li, Xiaopeng Guo, Yu Wang, Wenbin Ma

**Affiliations:** ^1^ Department of Neurosurgery, Peking Union Medical College Hospital Chinese Academy of Medical Sciences and Peking Union Medical College Beijing China

**Keywords:** glioblastoma, histone post‐translational modifications, O^6^‐methylguanine‐DNA methyltransferase, temozolomide resistance, transcription factor

## Abstract

**Backgroud:**

Glioblastoma multiforme (GBM) is among the most aggressive cancers, with current treatments limited in efficacy. A significant hurdle in the treatment of GBM is the resistance to the chemotherapeutic agent temozolomide (TMZ). The methylation status of the MGMT promoter has been implicated as a critical biomarker of response to TMZ.

**Methods:**

To explore the mechanisms underlying resistance, we developed two TMZ‐resistant GBM cell lines through a gradual increase in TMZ exposure. Transcriptome sequencing of TMZ‐resistant cell lines revealed that alterations in histone post‐translational modifications might be instrumental in conferring TMZ resistance. Subsequently, multi‐omics analysis suggests a strong association between histone H3 lysine 9 acetylation (H3K9ac) levels and TMZ resistance.

**Results:**

We observed a significant correlation between the expression of H3K9ac and MGMT, particularly in the unmethylated MGMT promoter samples. More importantly, our findings suggest that H3K9ac may enhance MGMT transcription by facilitating the recruitment of the SP1 transcription factor to the MGMT transcription factor binding site. Additionally, by analyzing single‐cell transcriptomics data from matched primary and recurrent GBM tumors treated with TMZ, we modeled the molecular shifts occurring upon tumor recurrence. We also noted a reduction in tumor stem cell characteristics, accompanied by an increase in H3K9ac, SP1, and MGMT levels, underscoring the potential role of H3K9ac in tumor relapse following TMZ therapy.

**Conclusions:**

The increase in H3K9ac appears to enhance the recruitment of the transcription factor SP1 to its binding sites within the MGMT locus, consequently upregulating MGMT expression and driving TMZ resistance in GBM.

## Graphical abstract

The acetylation of histone H3 at lysine 9 leads to an open chromatin structure at the MGMT gene locus, facilitating the binding of transcription factors SP1 to the promoter and enhancer regions, resulting in increased transcription of MGMT, thereby contributing to the TMZ resistance in GBM cells.

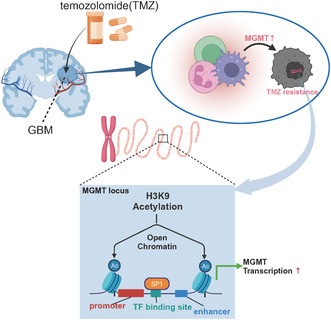

## 1 INTRODUCTION

Glioblastoma multiforme (GBM) stands as a formidable malignancy within the central nervous system,^1^, ^2^ characterized by rapid progression and marked resistance to therapeutic interventions, particularly to the chemotherapeutic agent temozolomide (TMZ).^3^, ^4^ GBM poses significant treatment hurdles. The O6‐methylguanine‐DNA methyltransferase (MGMT) gene is pivotal in this context.^5^, ^6^ Acting as a crucial DNA repair enzyme, MGMT mitigates the efficacy of TMZ by repairing the DNA modifications induced by the drug, thus diminishing its therapeutic impact.^7^, ^8^ The expression of MGMT is often silenced due to promoter hypermethylation, which is associated with a heightened response to TMZ.^6^, ^9^ In contrast, an unmethylated MGMT promoter typically indicates resistance to TMZ, aligning with poorer patient outcomes.^10^

However, the resistance of GBM to TMZ extends beyond the methylation status of the MGMT promoter.^11^, ^12^ The broader scope of epigenetic mechanisms, particularly histone post‐translational modifications (PTMs), plays an increasingly recognized role in the context of GBM resistance.^13^, ^14^, ^15^ Histones, the core proteins around which DNA is wound, undergo various PTMs that crucially shape the chromatin landscape,^16^ thereby influencing the accessibility of DNA for transcriptional activity.^17^, ^18^ Notably, some histone PTMs have been implicated in mediating TMZ resistance in GBM.^13^, ^19^ For example, while histone H3 lysine 27 trimethylation (H3K27me3) is associated with the silencing of tumor suppressor genes,^20^, ^21^ histone acetylation, specifically H3K27 acetylation (H3K27ac), has been linked to enhanced MGMT expression and consequently increased resistance to TMZ, irrespective of the promoter's methylation status.^22^

Grasping the intricate interplay between histone PTMs and TMZ resistance could be transformative for GBM treatment strategies. Our study delves deep into the nexus between specific histone PTMs and TMZ resistance in GBM. We aim to elucidate the complex interactions between these histone PTMs and MGMT, including the underlying mechanics of potential TMZ resistance. This comprehensive investigation aspires to overcome the resistance of TMZ treatment and refine therapeutic approaches for GBM, potentially paving the way for more effective and targeted treatments.

## 2 MATERIALS AND METHODS

### 2.1 Data collection and processing

We extracted transcriptomic profiles of normal brain tissues from diverse anatomical regions from the Genotype‐Tissue Expression (GTEx) database. Additionally, for IDH‐wildtype glioblastoma multiforme (GBM), we sourced transcriptomic datasets accompanied by clinical and follow‐up data from The Cancer Genome Atlas (TCGA) and the Chinese Glioma Genome Atlas (CGGA). Cancer Cell Line Encyclopedia (CCLE) database served as our reference for data on GBM cell lines. Single‐cell mRNA sequencing data specific to GBM tissues were retrieved from the GSE131298_10X dataset in the Gene Expression Omnibus (GEO) database. Furthermore, the GSE174554 dataset provided single‐nucleus transcriptomic data of both primary and recurrent GBM. To investigate the epigenetic and transcriptional shifts associated with TMZ resistance in GBM cells, we accessed the GSE113816 dataset in the GEO database, which encompasses chromatin immunoprecipitation sequencing (ChIP‐seq) and RNA sequencing (RNA‐seq) data for TMZ‐resistant and sensitive cell lines. Data visualization was executed using Integrative Genomics Viewer (IGV) software, annotating regulatory DNA elements like enhancers and promoters with BED files available in the IGV toolkit.

### 2.2 Single‐sample gene set enrichment analysis (ssGSEA)

The ssGSEA was employed for quantitative pathway assessment associated with histone PTMs. Relevant gene sets, closely linked to histone modifications, were extracted from the Molecular Signatures Database (MSigDB). The analysis was conducted using the GSVA^23^ package in R.

### 2.3 Machine learning methods

For optimal variable selection in predicting IC50, we harnessed a comprehensive machine learning framework. Our strategy incorporated three principal algorithms: generalized linear model (GLM), random forest, and support vector machine (SVM), leveraging the capabilities of the caret^24^ package in R.

### 2.4 Weighted gene co‐expression network analysis (WGCNA)

Employing the WGCNA^25^ package in R, we constructed a weighted gene co‐expression network. A pivotal step in our analysis was selecting the appropriate soft‐thresholding power (*β*) to ensure a scale‐free network topology. We then developed an adjacency matrix, subsequently transformed into the topological overlap matrix (TOM). Utilizing hierarchical clustering on the TOM, we identified modules of correlated genes using the dynamic tree cut method. For each module, the module eigengene (ME) was calculated, enabling a deeper understanding of gene co‐expression patterns.

### 2.5 Functional analysis

For a holistic insight into the overarching biological processes, Gene Set Enrichment Analysis (GSEA) was conducted using the GSEA v4.3.2. To elucidate the biological attributes and signaling pathways of our gene list, GO and Kyoto Encyclopedia of Genes and Genomes (KEGG) analysis was performed.

### 2.6 Transcriptome sequencing and proteomics analysis of TMZ‐resistant and parental GBM cell lines

For transcriptome sequencing, RNA from TMZ‐resistant and parental GBM cell lines was extracted using TRIzol Reagent (Invitrogen) and sequenced on an Illumina HiSeq platform. This procedure identified differentially expressed genes between these cell populations. Concurrently, proteomic analysis was conducted. Proteins were extracted, digested, and analyzed using tandem mass spectrometry (MS/MS). The resulting mass spectra were processed and matched to a protein database, identifying proteins with varied abundance between the TMZ‐resistant and original GBM cell lines.

### 2.7 Prediction of transcription factors targeting MGMT

Utilizing the TRRUST online database (https://www.grnpedia.org/trrust/), potential transcription factors interacting with MGMT were identified. These interactions were visualized using Cytoscape software. Further investigation into transcription factor binding sites on MGMT was conducted using the JASPAR online tool (https://jaspar.genereg.net/).

### 2.8 Single‐cell data and pseudotemporal analysis

Single‐cell transcriptome datasets for GBM were obtained from the TISCH2 database (http://tisch.comp‐genomics.org). Comprehensive visualization and analysis of GBM at a single‐cell resolution were conducted using the Seurat^26^ R package. To explore the developmental trajectory and progression dynamics of GBM cells, pseudotemporal ordering was applied using the Monocle^27^ R package.

### 2.9 Cell culture and establishment of TMZ‐resistant cell lines

U251 and U118 glioblastoma cell lines were obtained from the Institute of Biochemistry and Cell Biology, Chinese Academy of Science. Cell line authentication involved short tandem repeat (STR) profiling. Cells were cultured in Dulbecco's Modified Eagle Medium (DMEM), supplemented with 10% fetal bovine serum (FBS) and 1% penicillin‐streptomycin antibiotics (Gibco). Cultivation occurred in a humidified incubator at 37°C and 5% CO2. Routine mycoplasma testing confirmed the absence of contamination. To induce temozolomide resistance, cells from both U118 and U251 lines were split into independent replicates for parallel treatment. TMZ resistance was induced in U118 and U251 cells through incremental TMZ (Sigma, T2577) exposure, starting at 16 μg/mL and gradually increasing to 128 μg/mL over several months.^3^ Control cultures for both cell lines were maintained under similar conditions minus TMZ exposure.

### 2.10 MTT assay

The viability and TMZ resistance of GBM cell lines were meticulously assessed using the MTT proliferation assay. Cells were seeded in 96‐well plates and treated with varying concentrations of TMZ. Post‐incubation, MTT reagent was added to quantify cell viability through the conversion of MTT to formazan by metabolically active cells. Absorbance was measured at 490 nm using a microplate reader.

### 2.11 Quantitative real‐time PCR (qPCR) analysis

Relative expression of MGMT and SP1 genes in TR and NC cell groups was quantified using qPCR. Specific primers for MGMT (forward: 5'‐ACCGTTTGCGACTTGGTACTT‐3′ and reverse: 5'‐GGAGCTTTATTTCGTGCAGACC‐3′) and SP1 (forward: 5'‐TGGCAGCAGTACCAATGGC‐3′ and reverse: 5'‐CCAGGTAGTCCTGTCAGAACTT‐3′) were used. Expression levels were normalized to GAPDH and analyzed by the ΔΔCt method.

### 2.12 Western blot analysis

Proteins were extracted using RIPA lysis buffer containing protease and phosphatase inhibitors. Protein concentration was determined using the BCA Protein Assay Kit (Thermo Fisher Scientific). Approximately 30 μg of protein per sample was loaded and separated on a 15% SDS‐PAGE gel and transferred onto PVDF membranes. The membranes were blocked with 5% non‐fat milk in TBST for 1 h at room temperature and then incubated with primary antibodies overnight at 4°C, MGMT (ab39253, Abcam) and H3K9ac (ab32129, Abcam). After washing, the membranes were incubated with HRP‐conjugated secondary antibodies for 1 h at room temperature. Protein bands were visualized using the ECL Western Blotting Detection System (GE Healthcare).

### 2.13 Immunofluorescence staining

NC and TR cells were seeded onto coverslips placed in 6‐well plates. Cells were washed with PBS and fixed with 4% paraformaldehyde for 15 min at room temperature. After washing with PBS, cells were permeabilized with 0.1% Triton X‐100 and 0.05% NP‐40 in PBS for 10 min. Blocking was performed with 5% BSA in PBS for 1 h at room temperature. Cells were then incubated with the primary antibody overnight at 4°C, followed by incubation with a fluorochrome‐conjugated secondary antibody for 1 h in the dark at room temperature. Coverslips were mounted onto slides using mounting medium with DAPI. Images were captured using a fluorescence microscope.

### 2.14 ChIP‐qPCR Analysis

To investigate H3K9ac modification and SP1 binding at the MGMT locus, ChIP‐qPCR was performed on NC and TR cells. Cells underwent formaldehyde crosslinking, followed by lysis and chromatin fragmentation. The fragmented chromatin was immunoprecipitated using specific antibodies against MGMT, H3K9ac, and SP1. An anti‐IgG antibody was used as a negative control. Purified DNA was subjected to qPCR amplification using primers targeting MGMT TF binding sites. The fold enrichment of H3K9ac and SP1 at these sites was calculated relative to input DNA, comparing between NC and TR cell lines. The isolated DNA fragments were amplified by qPCR using MGMT TF binding site‐specific primers: forward 5'‐ACCTACGCTGTTAGCTCTGC‐3′ and reverse 5'‐TGCAGCCCTCCGATATAAGC‐3′.

### 2.15 Statistical analysis

We first assessed data distribution using Shapiro‐Wilk and Kolmogorov‐Smirnov tests for normality. For normally distributed data, Student's *t*‐test and one‐way ANOVA were applied for two‐group and multi‐group comparisons, respectively. Non‐normally distributed data were analyzed using Spearman's rank correlation and the Wilcoxon rank‐sum test. Kaplan‐Meier analysis with the log‐rank test assessed survival outcomes. All statistical tests were two‐sided with a *p*‐value threshold of <0.05, and data visualization was performed using ggplot2 in R.

## 3 RESULTS

### 3.1 Histone modification profiling in glioblastoma multiforme

Our transcriptomic analysis compared TMZ‐resistant U251‐TR and U118‐TR GBM cell lines to their parental counterparts. We identified a total of 692 upregulated and 118 downregulated genes in these cell lines, with a overlap in gene expression changes (Figure S1A–D). Functional analysis revealed a significant enrichment in histone modification and chromatin organization‐related genes among the upregulated set, whereas downregulated genes were mostly involved in retinoid metabolism (Figure S1E,F).

**FIGURE 1:**
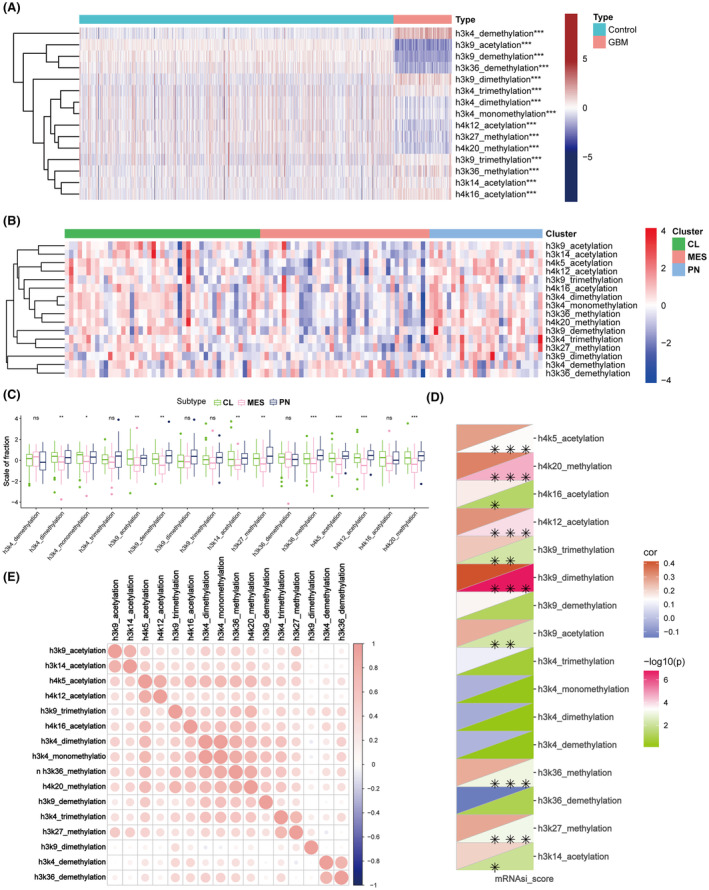
Landscape of Histone Post‐translational Modifications in GBM Samples. (A) Comparative visualization of active histone modifications among GBM and normal tissues. (B,C) Analysis of histone modification activities across GBM subtypes. (D) Heatmap of correlation between histone modification activities and GBM stemness scores. (E) Interrelationship matrix among various histone modifications.

Applying ssGSEA, we assessed histone PTM pathways across GBM and normal brain tissues. GBM tissues, particularly IDH‐wildtype samples from TCGA‐GBM and CGGA‐GBM datasets, exhibited significant different level of histone PTMs compared to normal brain tissues (Figure 1A). Specific histone modification activities, such as H3K4 dimethylation and H3K9 acetylation, varied across GBM subtypes (Figure 1B,C, *p* < 0.05). Spearman analysis highlighted the correlation among histone modification pathway activities and stemness of GBM (Figure 1D). Furthermore, inter‐correlations among various histone modifications suggested a synergistic network, exemplified by associations like H3K9 acetylation with H3K14 acetylation (Figure 1E). These findings collectively underscore the intricate landscape of histone modifications in the context of GBM, particularly in TMZ‐resistant strains.

### 3.2 Histone PTMs correlation with TMZ resistance in GBM

**FIGURE 2:**
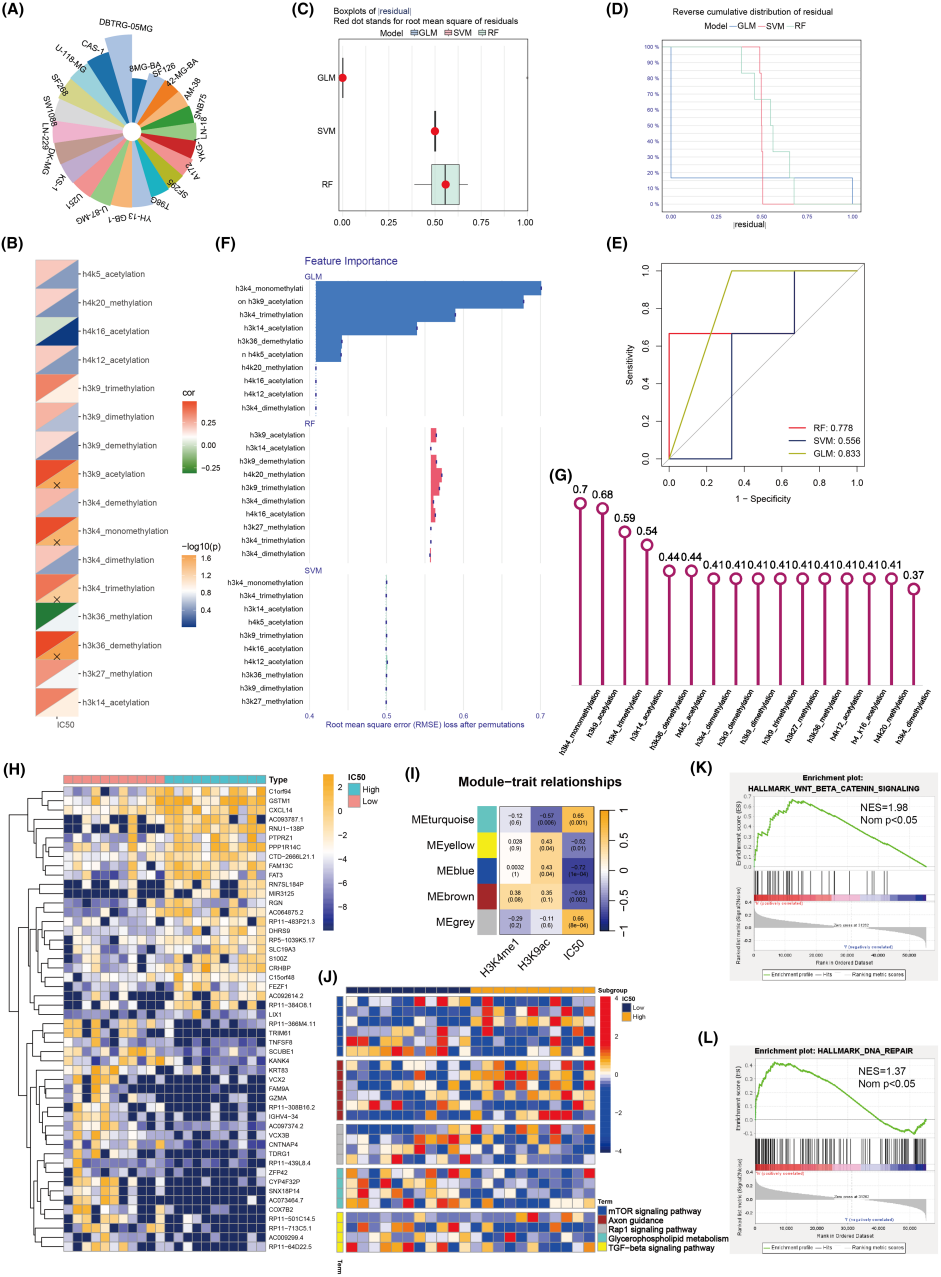
Exploration of Histone PTMs Correlation with TMZ Resistance in GBM Cell Lines from CCLE. (A) GBM cell lines ranked by TMZ IC50 values. (B) Histone modifications correlating with IC50. (C,D) Residual plots for machine learning model predictions. (E) ROC curve comparison of predictive models. (F) Importance of histone PTMs in SVM, GLM, and RF models for predicting TMZ IC50. (G) Importance of various histone PTMs on the basis of the application of the GLM algorithm. (H) Heatmap of differential gene expression across different IC50 subgroup. (I) Heatmap displays the correlation of gene modules associated with IC50, as identified by WGCNA, with H3K4me1 and H3K9ac. (J) KEGG pathway analysis annotated the potential biological functions implicated by different gene modules. (K,L) GSEA of H3K9ac in GBM samples, indicating the WNT‐beta catenin signaling and DNA repair pathway correlated with H3K9ac.

To investigate the correlation between histone PTMs and TMZ resistance in GBM, transcriptomic data and corresponding TMZ IC50 values from GBM cell lines in the CCLE database were analyzed. GBM cells were ranked by ascending IC50 values, and ssGSEA scores for various histone PTMs were computed (Figure 2A). Using a median IC50 cut‐off of 7.08 μM, cell lines were divided into high IC50 (TMZ‐resistant) and low IC50 (TMZ‐sensitive) groups. Spearman correlation highlighted pathways like H3K36 demethylation, H3K4 trimethylation, and H3K9 acetylation strongly correlating with IC50 (Figure 2B).

Three machine learning algorithms, including GLM, random forest, and SVM, were utilized to predict IC50 values based on histone PTMs. GLM demonstrated superior performance, indicated by lower residuals (Figure 2C,D) and the highest AUC of 0.833 (Figure 2E). Feature importance analysis identified H3K4me and H3K9ac as critical predictors in GLM (Figure 2F), with significant predictive scores (Figure 2G).

Differential gene expression analysis between the IC50 subgroups revealed potential downstream targets influenced by PTMs like H3K4me1 and H3K9ac (Figure 2H). Optimal soft power value was determined to be 6 based on scale independence and mean connectivity (Figure S2A,B). WGCNA identified gene modules correlated with H3K4me1, H3K9ac, and IC50, clustering genes into five modules (Figure S2C). Correlation heatmap showed significant associations between modules and IC50, particularly METurquoise, MEYellow, and MEBlue with H3K9ac (Figure 2I, Figure S2D–I). KEGG analysis revealed that the genes within these modules might be involved in biological pathways, such as the mTOR and TGF‐beta signaling pathways. (Figure 2J). GSEA revealed enrichment of “WNT beta‐catenin signaling” and “DNA repair” pathways in high H3K9ac activity GBM samples (Figure 2K,L, Figure S2J–O). This analysis underscores the impact of histone PTMs, especially H3K9ac, on TMZ resistance in GBM.

### 3.3 Interplay between H3K9ac and MGMT expression in GBM

**FIGURE 3:**
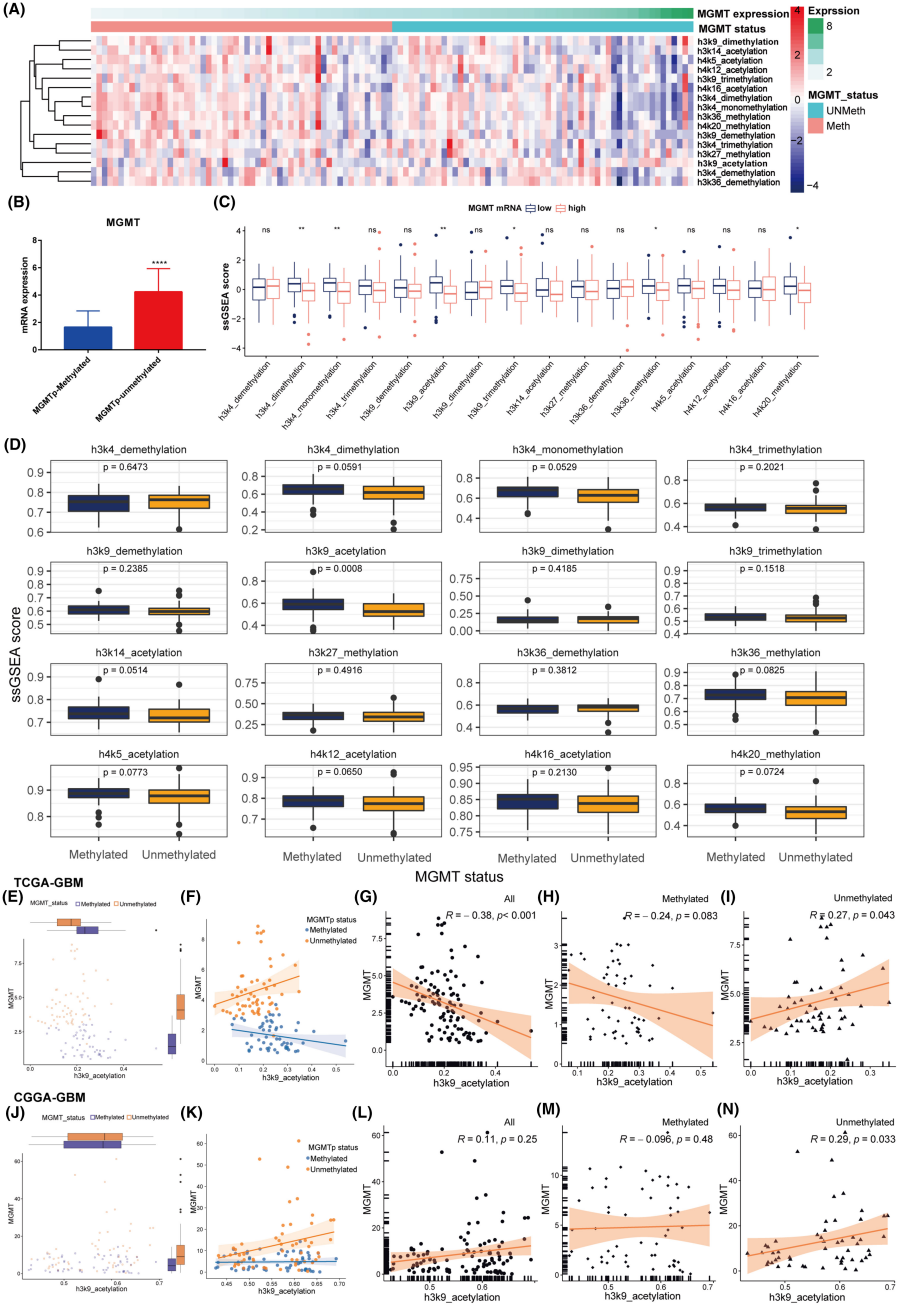
Interplay between Histone PTMs and MGMT Expression in Relation to Promoter Methylation. (A) Overview of MGMT expression levels vis‐a‐vis histone PTMs based on promoter methylation status. (B–D) Correlation analyses between methylation status, MGMT expression, and histone PTMs. (E–G) Analyses on the correlation between H3K9ac and MGMT expression in TCGA‐GBM dataset samples in different MGMT promoter methylation status samples. Correlation between MGMT expression and H3K9ac activity in (H) methylated and (I) non‐methylated MGMT promoters. (J–N) Validation from the CGGA‐GBM dataset.

In our investigation of TMZ resistance in GBM, we focused on the relationship between histone PTMs and MGMT expression, particularly considering MGMT promoter methylation status. Figure 3A displays a heatmap contrasting MGMT expression with histone PTMs, categorized by MGMT promoter methylation status. We observed lower MGMT mRNA levels in samples with methylated MGMT promoter (Figure 3B). Further, significant variations in PTMs like H3K4me2 and H3K9ac were noted, especially in low MGMT expression subgroups (Figure 3C).

Exploring the link between histone PTMs and MGMT promoter methylation (Figure 3D), we found a significant increase of H3K9ac in the methylated MGMT promoter subgroup (p = 0.0008), suggesting an inverse relationship with MGMT expression. Meanwhile, analysis of the TCGA‐GBM dataset revealed a distinct negative correlation between MGMT expression and H3K9ac activity (R = −0.38, *p* < 0.05) across the dataset (Figure 3E–G). This correlation was absent in the methylated subgroup (Figure 3H) but showed a significant positive correlation among MGMT and H3K9ac in the unmethylated subgroup (Figure 3I).

These results were supported by the CGGA‐GBM dataset, showing a positive correlation between MGMT expression and H3K9ac (R = 0.29, *p* < 0.05) in the non‐methylated MGMT promoter subgroup (Figure 3J–N). This finding implies that in the absence of MGMT promoter methylation, elevated H3K9ac levels may promote MGMT expression in GBM. However, this apparent significant positive correlation may be obscured by the methylation of the MGMT promoter. In other words, the silencing of MGMT expression due to its promoter methylation could potentially interfere with the observed correlation.

### 3.4 Impact of MGMT promoter methylation and H3K9ac on prognosis of GBM

**FIGURE 4:**
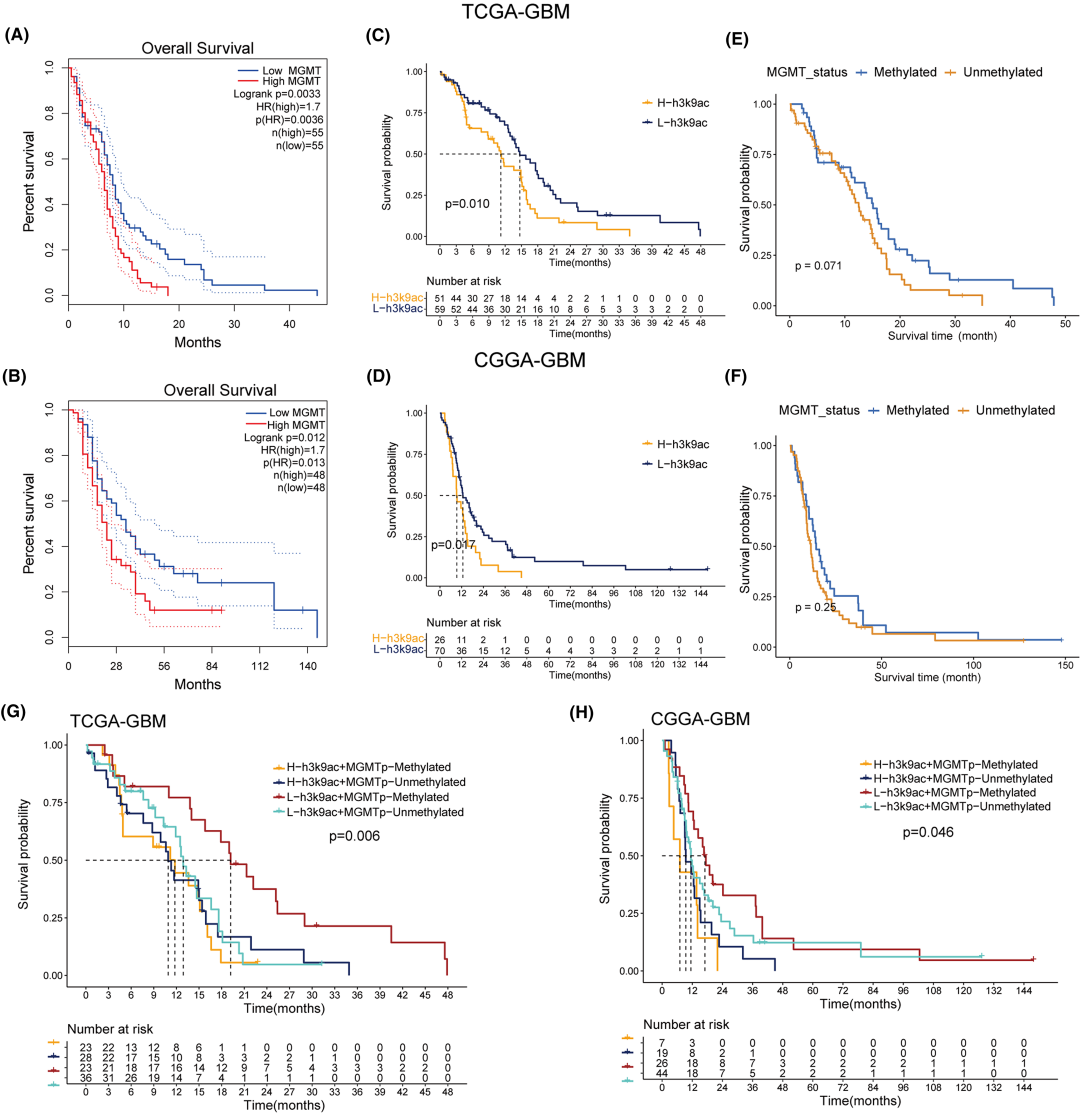
Prognostic Relevance of MGMT Expression and H3K9ac Levels in GBM Patients Treated with TMZ. (A–F) Kaplan‐Meier survival analyses based on MGMT expression, H3K9ac levels, and MGMT promoter status in TCGA and CGGA cohorts. (G–H) Combined analysis of MGMT methylation status and H3K9ac levels on prognosis of GBM.

In assessing TMZ treatment efficacy for GBM, we analyzed the prognostic significance of MGMT expression and H3K9ac levels in relation to MGMT promoter methylation. Higher MGMT expression was linked to decreased overall survival (OS) in both TCGA and CGGA‐GBM cohorts, as depicted in Figure 4A,B. Similarly, elevated H3K9ac levels corresponded with poorer outcomes, as shown in survival analyses (Figure 4C,D). Interestingly, MGMT promoter methylation status did not significantly stratify patient outcomes in these cohorts (Figure 4E,F).

A composite survival analysis combining both factors showed that GBM patients with lower H3K9ac and methylated MGMT promoter had the best OS among four groups (Figure 4G,H). Additionally, TCGA‐GBM and CGGA‐GBM data indicated that high H3K9ac levels, particularly in cases with MGMT promoter methylation, were associated with significantly poorer prognosis. Conversely, in the absence of MGMT promoter methylation, H3K9ac levels had a weaker impact on OS. The influence of MGMT promoter methylation on OS of GBM was more pronounced at low H3K9ac activity levels, while its impact diminished at higher H3K9ac levels (Figure 4G,H). These findings indicate that both MGMT expression and H3K9ac levels can prognosticate outcomes in GBM patients, with H3K9ac potentially surpassing the predictive ability of MGMT promoter methylation status. Combined survival analyses demonstrate that even in the presence of MGMT promoter methylation, the levels of H3K9ac may still significantly impact the prognosis of GBM patients undergoing TMZ treatment.

### 3.5 Regulatory dynamics of MGMT and H3K9ac in GBM's TMZ resistance

**FIGURE 5:**
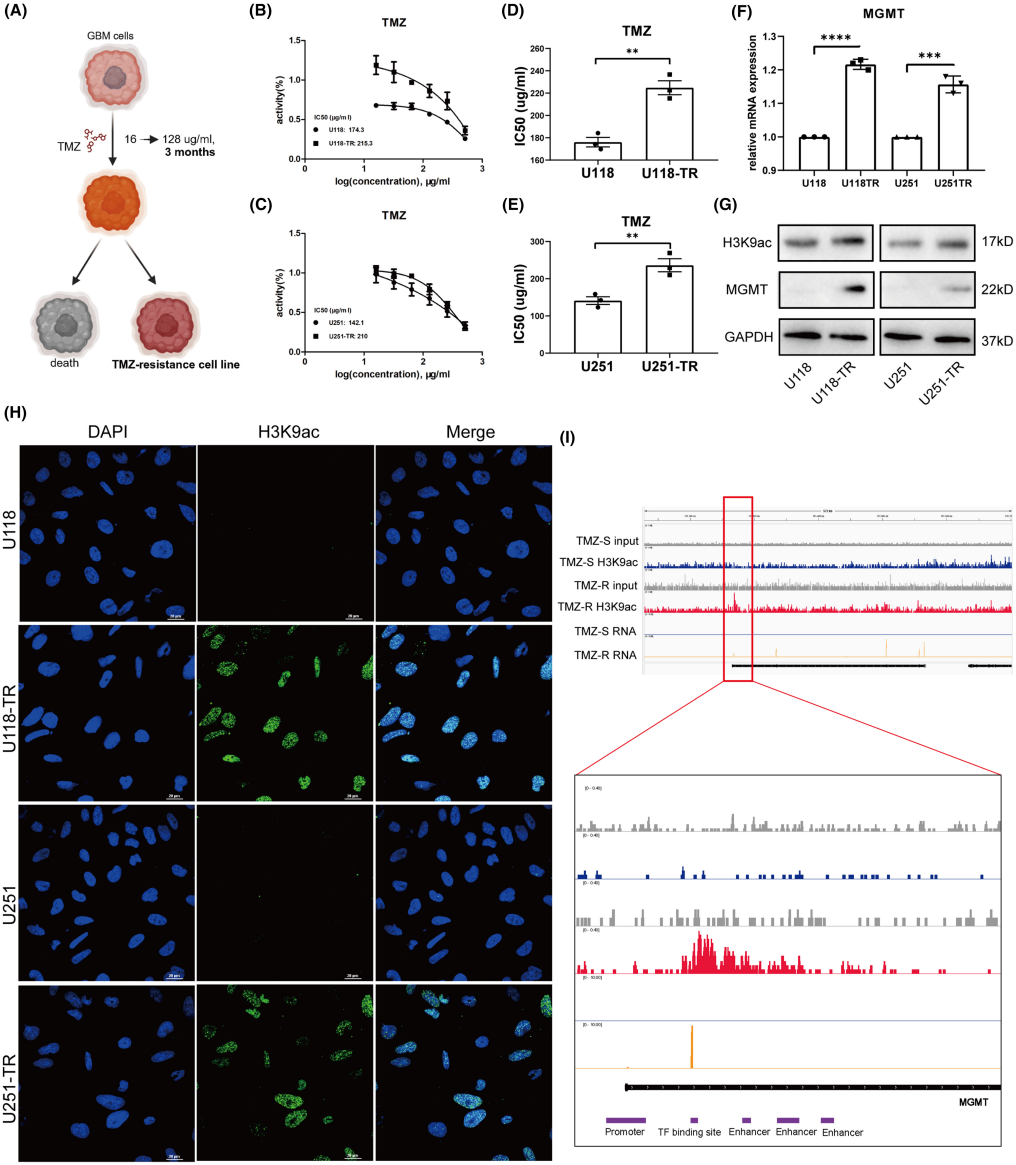
Interplay between MGMT Expression and H3K9ac in TMZ‐resistant GBM Cell Lines. (A) Workflow for developing TMZ‐resistant cell lines. (B,C) Response of NC‐ and TR‐cell lines to TMZ treatment. (D‐E) Boxplot of TMZ IC50 among U118, U118‐TR, U251, and U251‐TR cells. (F–H) Relative MGMT mRNA expression, WB analysis of MGMT and H3K9ac, and cellular immunofluorescence assay of H3K9ac in U118, U118‐TR, U251, and U251‐TR cells. (I) IGV visualization of H3K9ac at the MGMT locus in TMZ‐sensitive and TMZ‐resistant cells. The chip‐seq and mRNA data were obtained from GSE113816.

To decipher the relationship between MGMT expression and H3K9 acetylation in GBM's TMZ resistance, we developed U118‐TR and U251‐TR TMZ‐resistant cell lines (Figure 5A). Chronic TMZ exposure led to significantly higher TMZ IC50 values in these resistant lines compared to their parental counterparts (Figure 5B–E). Analysis of MGMT mRNA in these lines showed marked upregulation in the resistant strains (Figure 5F), a finding mirrored at the protein level (Figure 5G). Nuclear protein extractions revealed increased H3K9ac in the resistant strains (Figure 5G, Figure S3A), and immunofluorescence analysis confirmed enhanced nuclear localization of H3K9ac (Figure 5H). ChIP‐seq and RNA‐seq data from the GSE113816 dataset (Figure S3B) showed a significant elevation of H3K9ac at the MGMT locus in resistant strains, linked with higher MGMT transcription (Figure 5I). This upregulation was especially notable at promoter‐proximal transcription factor binding sites and enhancer regions, indicating H3K9ac's role in promoting chromatin accessibility and transcription factor binding.

Conversely, histone PTMs in DNA regions of other TMZ resistance‐associated genes like ATRX, MPG, and PARP1 did not exhibit similar H3K9ac upregulation (Figure S3C), pointing to a selective regulatory mechanism where H3K9ac predominantly affects MGMT expression, contributing to TMZ resistance.

### 3.6 Role of SP1 and H3K9ac in MGMT regulation within TMZ‐resistant GBM Cells

**FIGURE 6:**
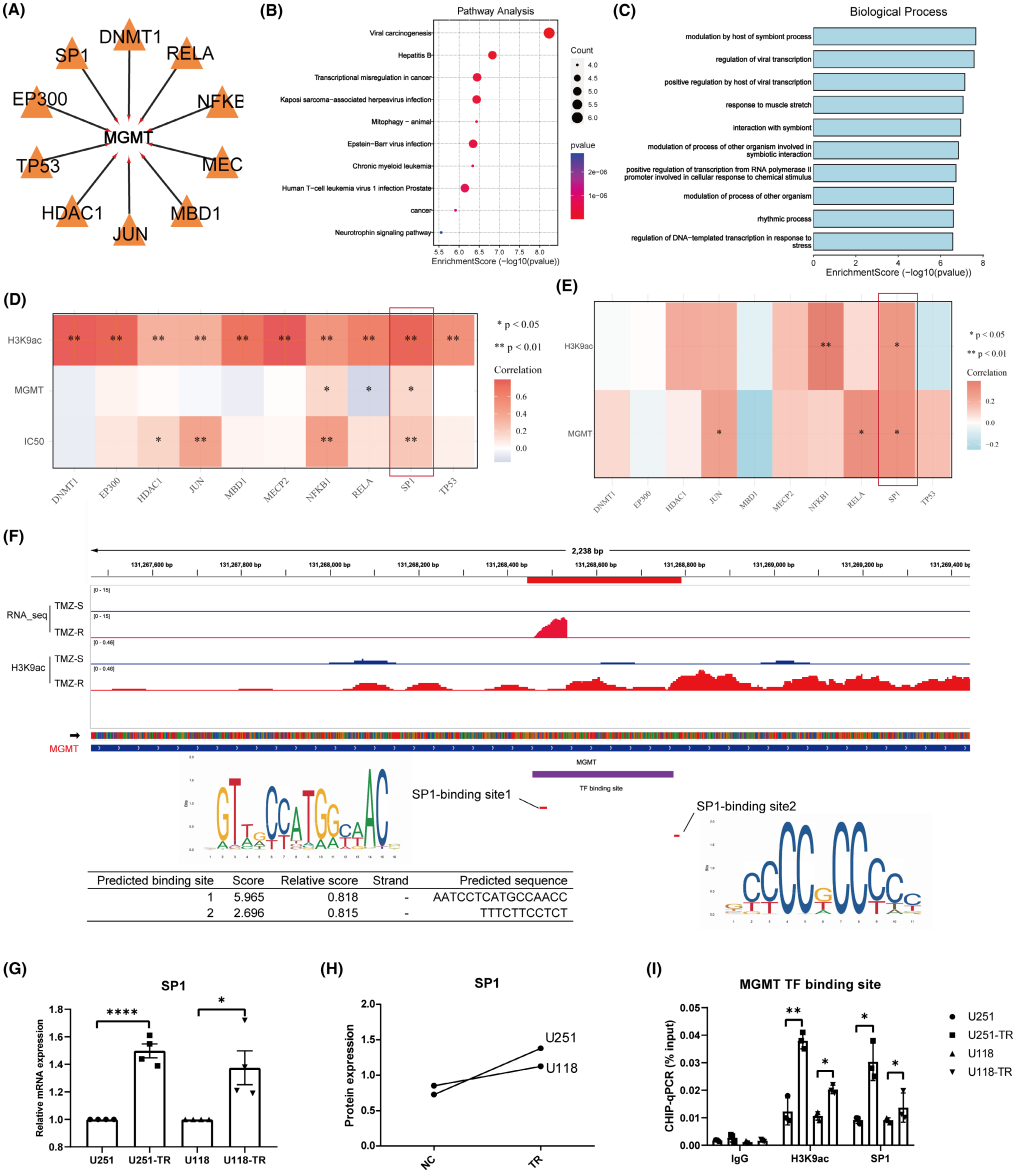
Elucidation of Transcription Factors and their Role in MGMT Regulation in GBM TMZ Resistance. (A) Cytoscape visualization of transcription factors targeting MGMT. (B‐C) KEGG and GO analysis of these TFs targeting MGMT potentially. (D,E) Heatmap demonstrating the correlation between the RNA expression levels of identified transcription factors, H3K9ac pathway activity, MGMT expression, and IC50 values in TCGA and CGGA‐GBM cohorts. (F) Depiction of predicted SP1 binding motifs and their sequences on MGMT from JASPAR, details of binding indices and relative scores for two main sites. (G,H) qPCR and mass spectrometry analyses confirming the upregulation of SP1 at RNA and protein levels, respectively, in the TMZ‐resistant cell lines. (I) Chip‐qPCR results highlighting significant enrichment of both H3K9ac and SP1 in the MGMT TF binding site region in the TMZ‐resistant cell strains.

In our exploration of MGMT transcriptional regulation in TMZ‐resistant GBM cells, we identified key transcription factors targeting MGMT, using the TRRUST database and Cytoscape network visualization (Figure 6A). Pathway analysis underscored the significant role of these transcription factors in various biological processes (Figure 6B,C). Analyzing correlations between transcription factors, H3K9ac, MGMT, and TMZ IC50 values, we found that SP1 uniquely correlated positively with H3K9ac, MGMT levels, and IC50 values in resistant cells (Figure 6D). This correlation was further supported by data from the CGGA‐GBM dataset, confirming SP1's association with H3K9ac and MGMT expression (Figure 6E). Investigating SP1's regulatory effect on MGMT, we predicted SP1 binding sites on the MGMT locus using JASPAR, identifying two potential binding motifs within high H3K9ac regions (Figure 6F). These sites demonstrated strong SP1 affinity within the TF binding region. We validated SP1 upregulation in resistant cell lines through qPCR and protein expression assays (Figure 6G,H). ChIP‐qPCR targeting the predicted SP1 binding sites confirmed significant enrichment of H3K9ac and SP1 at these loci in resistant strains (Figure 6I), suggesting that SP1, alongside H3K9ac, plays a pivotal role in upregulating MGMT, contributing to TMZ resistance development.

### 3.7 Dynamics of H3K9ac, SP1, and MGMT in GBM recurrence

**FIGURE 7:**
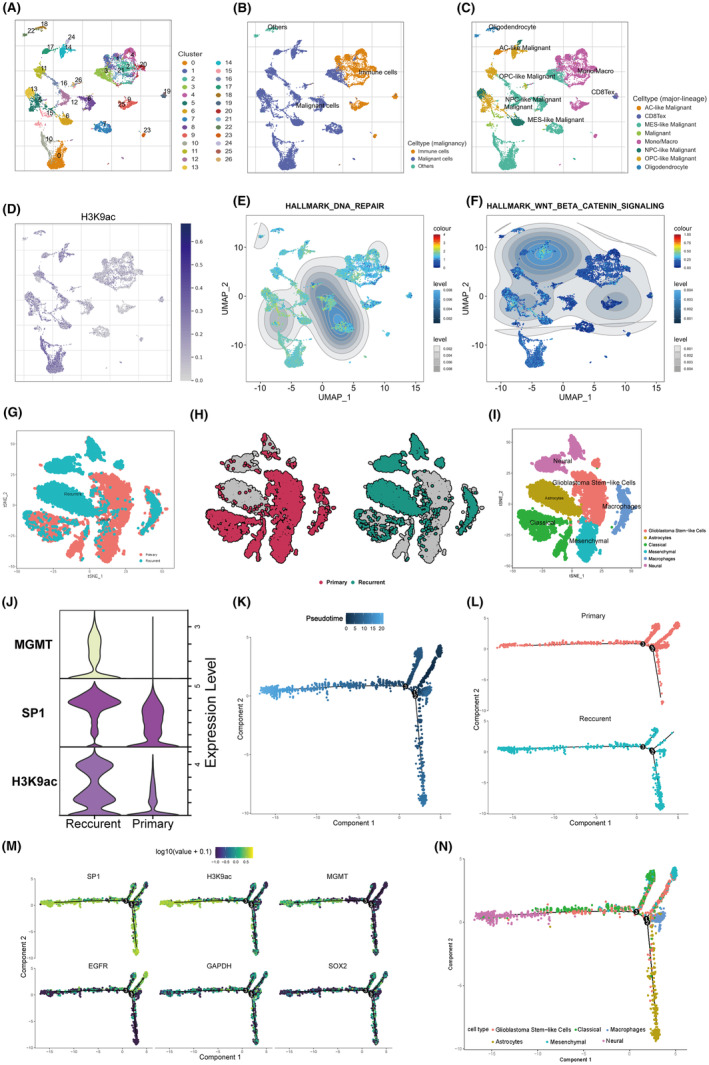
Single‐cell Transcriptomic Analysis of GBM Reveals Distinct Expression Patterns and Cellular Subtypes. (A) Visualization of 26 identified cell clusters from the GSE131298_10X dataset. (B) Annotation of the identified cells into malignant tumor cells, immune cells, and other cell types. (C) Subtyping of malignant tumor cells into NPC‐like, OPC‐like, AC‐like, and MES‐like categories. (D) Mapping of H3K9ac pathway activity on dimensionally reduced cells. (E,F) GSEA comparisons of the DNA‐repair and WNT‐β catenin pathway activities across cell subgroups. (G‐H) Dimensional reduction visualizations of different primary and paired recurrent GBM samples from the GSE174554 dataset. (I) Annotation of cellular subpopulations. (J) Comparative expression of H3K9ac, SP1, and MGMT between primary and recurrent samples. (K) Pseudotemporal analysis trajectories depicting primary and recurrent cells. (L) Concentration of primary and recurrent sample cells on pseudotime. (M) Expression trend of SP1, H3K9ac, MGMT, EGFR, GAPDH, and SOX2 on pseudotemporal. (N) Differentiation cell clusters of GBM on the location of pseudotime.

We then utilized single‐cell transcriptomic analysis to uncover the dynamics of H3K9ac, SP1, and MGMT across GBM subtypes. Analyzing the GSE131298_10X dataset comprising 9 GBM samples with 13,553 cells, we categorized cells into 26 distinct clusters (Figure 7A). These clusters included various cell types, notably malignant tumor cells, which were further subtyped into categories such as NPC‐like, OPC‐like, AC‐like, and MES‐like (Figure 7B,C). The distribution of these subtypes is depicted in Figure S4A. Mapping the expression of H3K9ac‐related genes allowed us to represent H3K9ac pathway activity on the dimensionally reduced cellular landscape (Figure 7D). Differential analysis with violin plots confirmed the upregulation of the H3K9ac pathway in malignant tumors (Figure S4B). Further, MES‐like cells exhibited marginally higher average levels of H3K9ac compared to other subtypes (Figure S4C). Given their known aggressive nature and resistance to chemotherapeutic interventions, the mesenchymal subtype's association with the H3K9ac pathway was intriguing. Through GSEA, we compared the activity of the DNA‐repair pathway and the WNT‐β catenin pathway (a previously identified potential downstream pathway, Figure 2K,L) across different cell subgroups (Figure 7E,F), indicating a stronger alignment of H3K9ac with the DNA‐repair pathway.

To delve into the expression dynamics of H3K9ac, SP1, and MGMT during tumor recurrence, paired primary and recurrent GBM samples from the GSE174554 dataset were studied. These patients underwent standard TMZ therapy but exhibited rapid relapse. Single‐cell transcriptomic data underwent rigorous quality control and preprocessing (Figure S4D). After selecting optimal PC numbers 7, (Figure S4E,F) and resolution (0.1, Figure S4G), eight clusters were identified (Figure S4H,I). Dimensional reduction visualized different primary and paired samples (Figure 7G,H). With references from the SingleR package (Figure S4J) and GBM subtype‐associated markers (Figure S4K), cellular annotations were established (Figure 7I). When comparing H3K9ac, SP1, and MGMT levels between primary and recurrent samples, all showed increased expression in the recurrent subgroups (Figure 7J). Pseudotemporal analysis (Figure 7K) depicted the trajectories of primary and recurrent cells over time, with primary samples primarily in early phases (Figure 7L). Earlier cell populations were characterized as glioblastoma stem‐like cells (Figure S4L). As tumor recurrence progressed and using GAPDH as a reference, the stemness of the tumor decreased, some cellular subpopulations persisted as mesenchymal GBM, followed by differentiation into astrocyte and classic types GBM, subsequently differentiating into neuronal type GBM, and MGMT, SP1, and H3K9ac levels trended upwards in this process (Figure S4L,M and Figure 7M,N). This comprehensive analysis highlights the intricate role of H3K9ac, SP1, and MGMT in GBM subtypes, particularly in the context of tumor recurrence and TMZ resistance.

## 4 DISCUSSION

Historically, research into TMZ resistance in GBM has lacked comprehensiveness, particularly in relation to the role of histone post‐translational modifications—an area of epigenetics that is crucial yet underexplored in the context of resistance mechanisms. This study firstly systematically investigated the potential association between histone PTMs and TMZ resistance and tried to delineate the underlying mechanisms. The methylation status of the MGMT promoter has long been underscored as a predictive marker for outcomes of TMZ therapy.^7^, ^28^ However, our recent findings suggest that MGMT promoter methylation does not always invariably confer a reliable prognostic value for therapeutic efficacy. The potential explanations for TMZ resistance seem to be rooted more profoundly in the expression levels of MGMT. While promoter methylation is a critical regulator of MGMT expression, it does not account for cases where patients manifest intrinsic or acquired resistance to TMZ despite the presence of promoter methylation. Intriguingly, our study indicates that histone PTMs, particularly the elevation of H3K9ac levels, could alter the transcriptional milieu of the MGMT gene, thereby upregulating MGMT expression and contributing to the mechanism of TMZ resistance. This has substantive clinical implications: Even patients with methylated MGMT promoters might exhibit TMZ resistance.^29^, ^30^ Therefore, a more accurate and comprehensive approach in clinical decision‐making would involve considering both the methylation status of the MGMT promoter and H3K9ac levels to predict a patient's potential benefit from TMZ treatment.

Equally important, the construction of a landscape of histone PTMs within GBM in our research offers direction for future in‐depth studies. This study also proposes potential strategies for the regulation of MGMT expression that are independent of DNA methylation. Leveraging multi‐omics data and our attempts to establish TMZ‐resistant cell lines, we have explored mechanisms by which H3K9ac may influence MGMT gene expression. H3K9ac, a common form of histone acetylation, is a crucial post‐translational modification that impacts chromatin structure and function.^31^ In terms of gene transcription, H3K9 acetylation is predominantly associated with transcriptional upregulation,^32^ leading to a more accessible chromatin configuration that facilitates transcription.^31^, ^33^ In our investigation, H3K9ac appears to support MGMT transcription by enhancing chromatin accessibility at the promoter region, allowing transcription factors such as specificity protein 1 (SP1)^34^ to bind more effectively to the TF binding sites on the MGMT locus.^35^ SP1, a ubiquitously expressed transcription factor, is known to regulate a myriad of genes controlling cellular processes, including growth, differentiation, and DNA damage response.^36^ The synergistic action of H3K9ac and SP1 activates MGMT transcription, thereby upregulating MGMT expression. This suggests the potential mechanism by which H3K9ac regulation of MGMT contributes to the acquired resistance of GBM cells to TMZ therapy.

Single‐cell transcriptomic analyses in this study found the positive correlation between the mesenchymal subtype, characterized by its chemotherapeutic resistance,^37^ and heightened H3K9ac pathway activity. It underscores the role of epigenetic modifications in tumor resistance, pointing toward novel therapeutic targets. Additionally, our pseudotemporal evaluations delineate the recurrence dynamics of GBM. This trajectory, marked by a shift from stem‐like features to a more differentiated state concomitant with elevated MGMT, SP1, and H3K9ac expressions, reveals a dynamic regulatory mechanism that could inform the development of more effective, personalized treatment strategies.

Our study, while offering valuable insights, is not without limitations. The use of cell culture models and reliance on public databases necessitate cautious interpretation and further validation to establish clinical relevance. A notable limitation is the lack of specific techniques to modulate H3K9ac modifications, highlighting the need for future research focused on targeted histone PTMs interventions through pharmacological agents, genetic editing, or other novel methods. Investigating the phenotypic and mechanistic changes resulting from such interventions is crucial. Additionally, while TMZ‐induced cell lines were used for validation, primary cells from GBM patients would provide a more accurate model.

In conclusion, we propose that increased levels of H3K9ac could be the potential driver behind TMZ resistance in GBM. This increase in H3K9ac appears to enhance the recruitment of the transcription factor SP1 to TF binding sites within the MGMT locus, consequently upregulating MGMT expression.

## AUTHOR CONTRIBUTIONS

LGY, LGG, and YNW contributed to the conception and design of this study. LGY, XPG, PTL, and HX contributed to the analysis and interpretation of data. WBM collaborated closely with YW in project oversight. All authors read and approved the final manuscript.

## FUNDING INFORMATION

This work was funded by the National High Level Hospital Clinical Research Funding (2022‐PUMCH‐A‐019), the CAMS Innovation Fund for Medical Sciences (2021‐I2M‐1‐014), the Beijing Municipal Natural Science Foundation (7202150, 19JCZDJC64200[Z]), the National Natural Science Foundation of China (82151302), the National High Level Hospital Clinical Research Funding (2022‐PUMCH‐B‐113), and the Tsinghua University‐Peking Union Medical College Hospital Initiative Scientific Research Program (2019ZLH101).

## CONFLICT OF INTEREST STATEMENT

The authors have no conflict of interest.

## Supporting information


Figures S1–S4.

## Data Availability

The datasets used and/or analyzed during this study are available from the corresponding author upon reasonable request.
