# Supplementary material for: Identification of TMZ resistance‐associated histone post‐translational modifications in glioblastoma using multi‐omics data
Source: CNS Neurosci Ther. 2024 Mar 6;30(3):e14649. doi: 10.1111/cns.14649 (PMC10917648; doi:10.1111/cns.14649)
Supplement: Supplementary file 1 — Figures S1–S4. [file CNS-30-e14649-s001.docx]

**Supporting information**


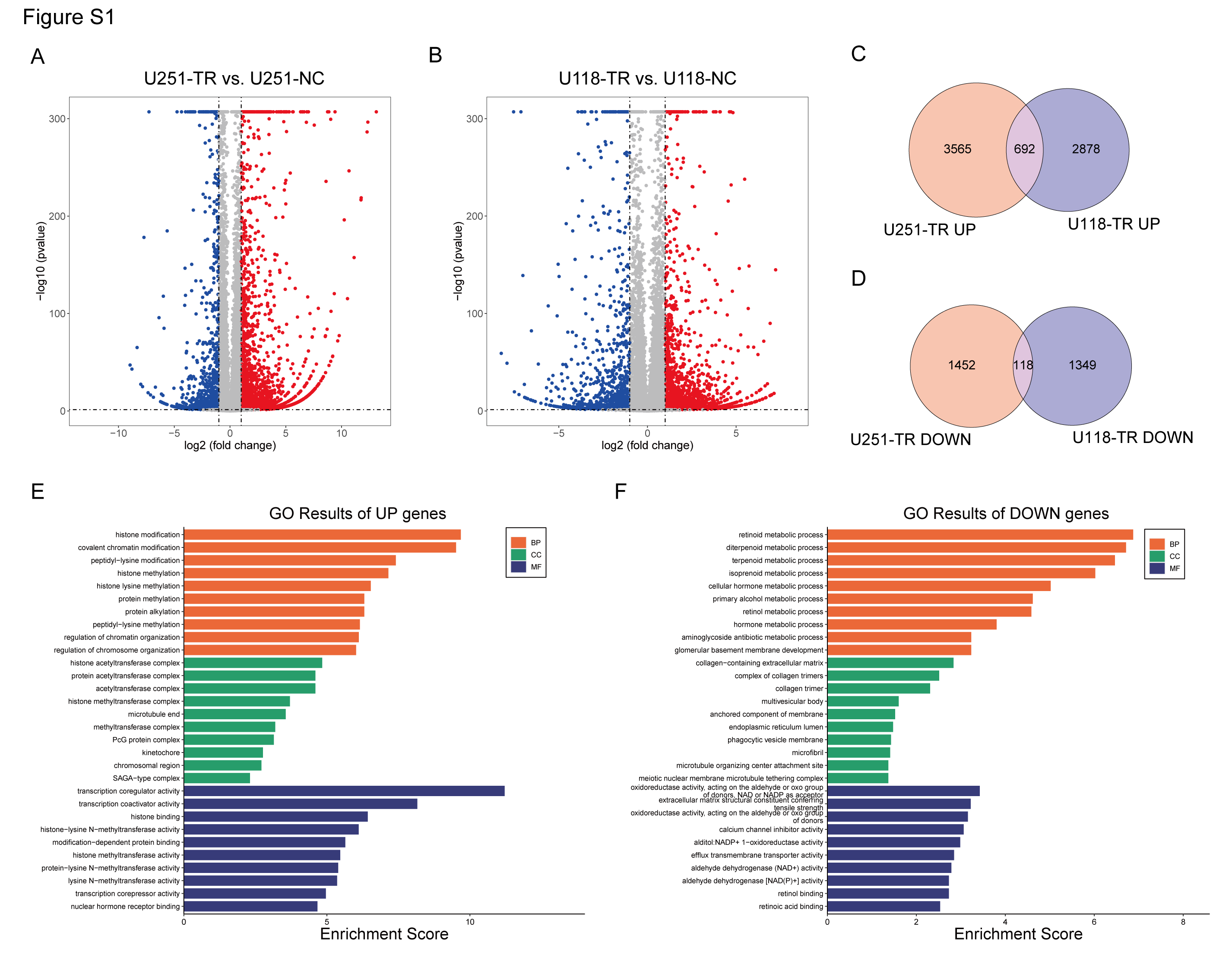


**Supplemental Figure 1. Analysis of Differentially Expressed Genes in Temozolomide-Resistant GBM Cell Lines.** (A, B) Volcano plot showcasing the differentially expressed genes in the U251-TR and U118-TR cell line. (C, D) Venn diagram depicting the intersecting genes that are commonly differentially expressed between U118 and U251 cell lines. (E, F) GO functional enrichment analysis of the intersecting upregulated and downregulated genes.


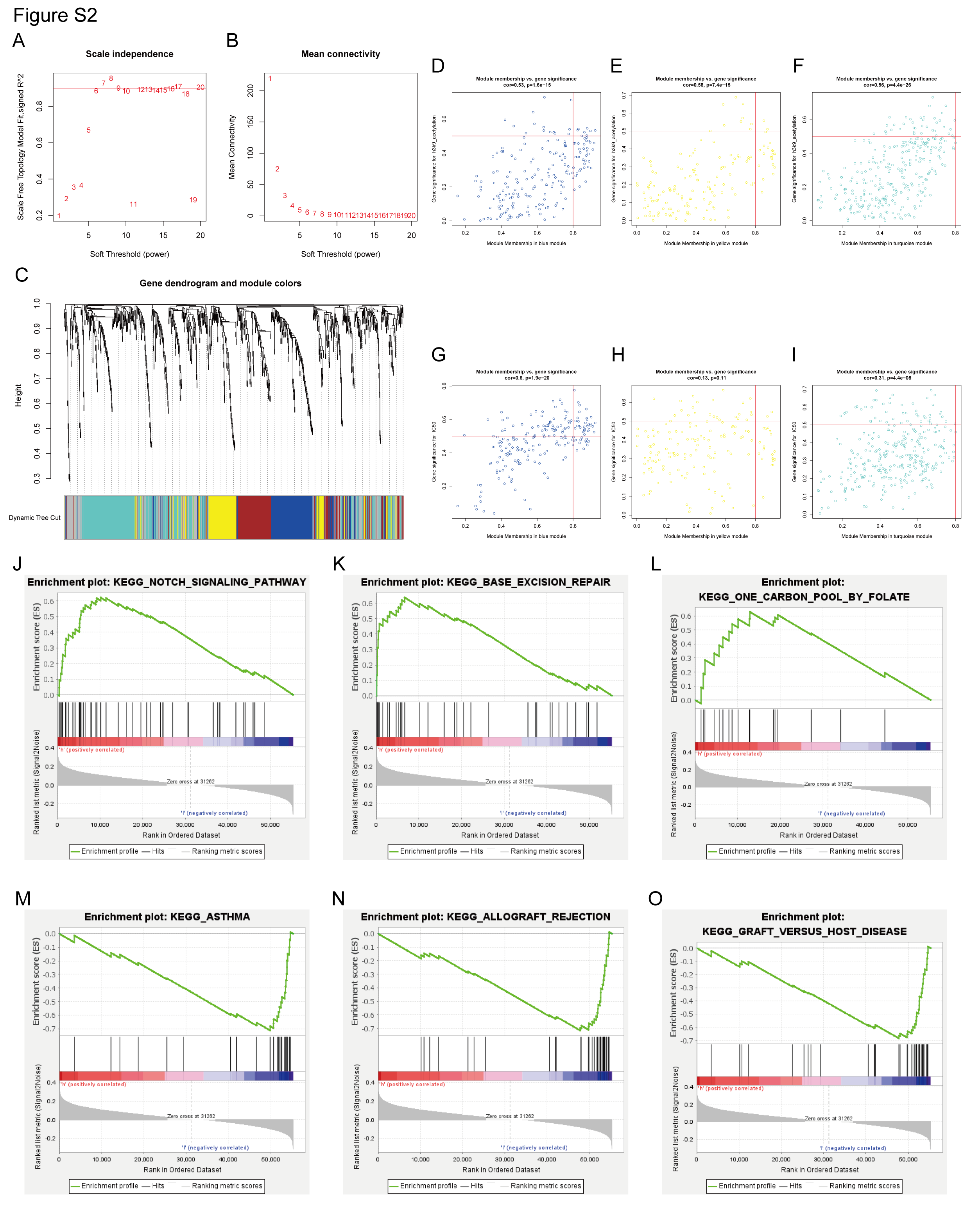


**Supplemental Figure 2. Gene Clustering and Pathway Enrichment Analysis in Relation to Histone PTMs and TMZ IC50 Values.** (A-B) Plots for scale independence and mean connectivity determining the optimal soft power value for WGCNA. (C) Dendrogram visualizing gene clustering resulting in five modules: turquoise, yellow, blue, brown, and grey. (D-I) Scatter plots correlating gene modules with h3k4me1, h3k9ac, and IC50. (J-O) Additional KEGG pathways of significance from the GSEA analysis related to H3K9ac in GBM cell lines.


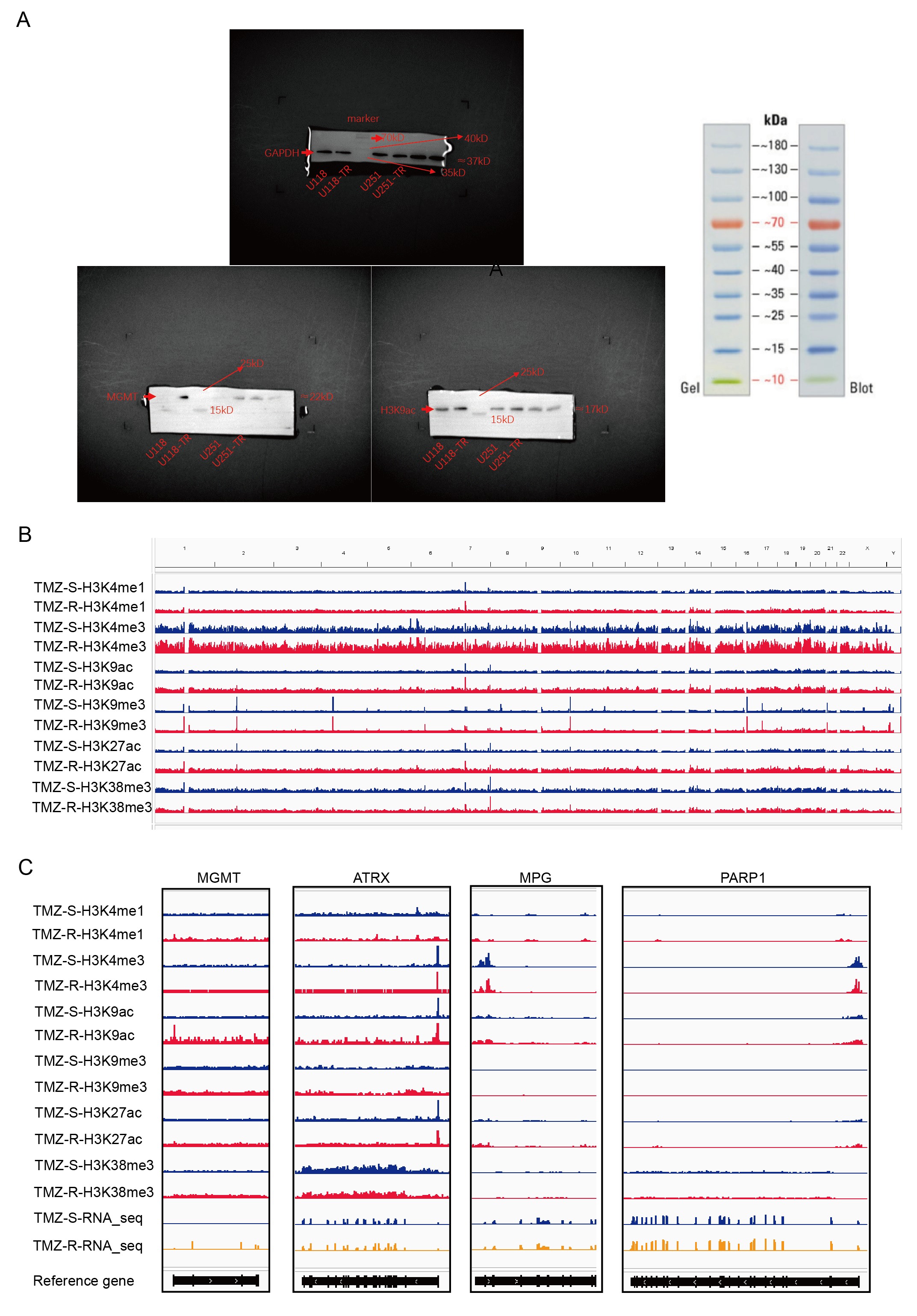


**Supplemental Figure 3. Histone Modifications and Expression Patterns of TMZ Resistance-Associated Genes in the GSE113816 Dataset.** (A) Overview of the chip-seq and corresponding RNA-seq data derived from the GSE113816 dataset available in the GEO database. (B) Visualization on the Integrative Genomics Viewer (IGV) of various TMZ resistance-associated genes, including MGMT, ATRX, MPG, and PARP1. The respective genomic regions of these genes are examined for their histone post-translational modifications, with a primary focus on H3K9ac modification.


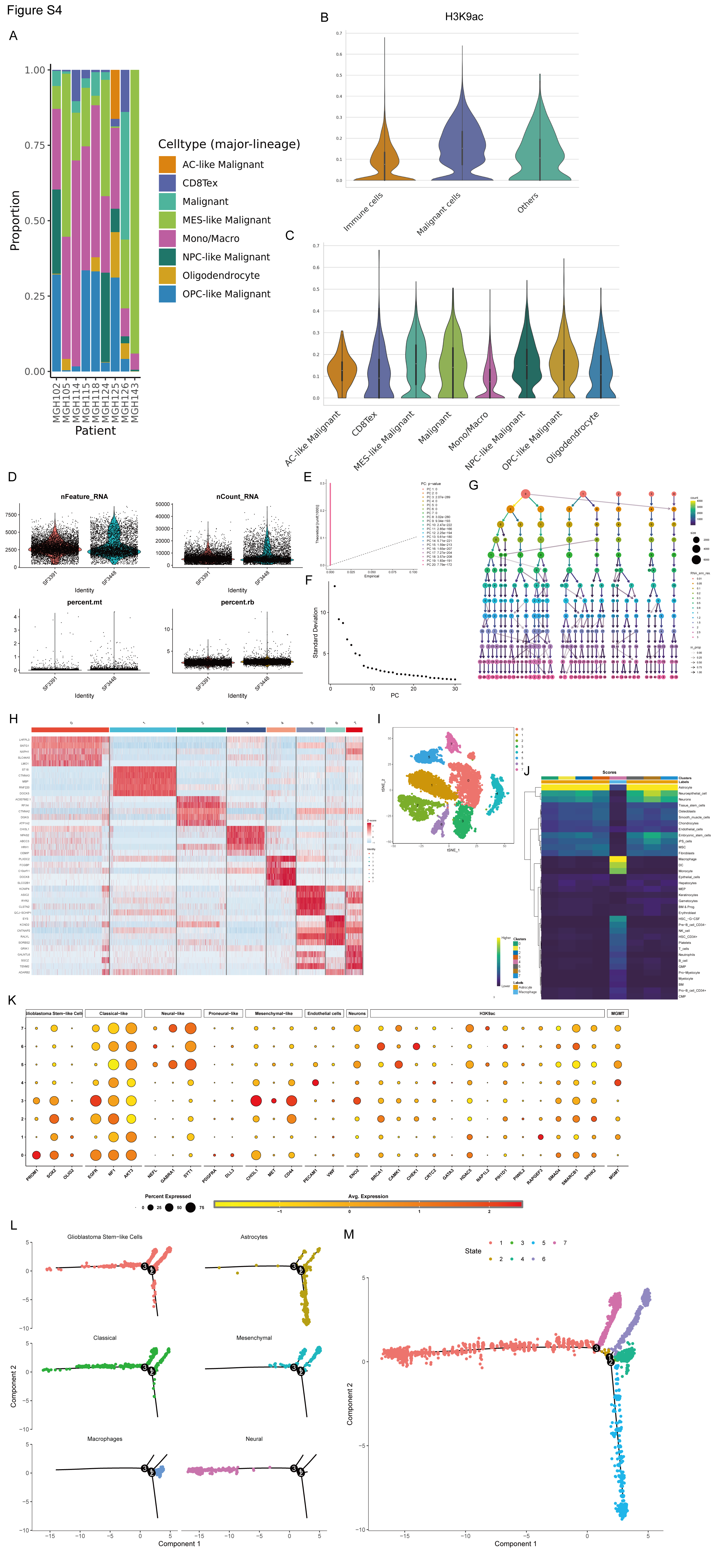


**Supplemental Figure 4. Supplementary Analysis and Visualization of GBM Single-cell Data.** (A) Proportional distribution of cellular subtypes across the nine GBM samples. (B) Violin plots highlighting the differential upregulation of the H3K9ac pathway in malignant tumor cells. (C) The distribution of H3K9ac levels in different GBM clusters. (D) Quality control and preprocessing steps for the single-cell transcriptomic data. (E-F) Optimal PC number selection. (G) Resolution optimization for cluster identification. (H-I) Identification and visualization of eight cell clusters. (J) Annotation of clusters using the singleR package. (K) GBM subtype-associated marker identification. (L) Characterization of cellular differentiation and subtype patterns throughout tumor progression. (M) Pseudotemporal analysis showing different states of cellular progression.
